# Offering non‐invasive prenatal testing as part of routine clinical service. Can high levels of informed choice be maintained?

**DOI:** 10.1002/pd.5154

**Published:** 2017-10-17

**Authors:** Celine Lewis, Melissa Hill, Lyn S. Chitty

**Affiliations:** ^1^ North East Thames Regional Genetics Service Great Ormond Street Hospital for Children NHS Foundation Trust London UK; ^2^ UCL Institute of Child Health and Great Ormond Street Institute of Child Health London UK

## Abstract

**Objectives:**

To assess rates of informed choice among women offered non‐invasive prenatal testing (NIPT) for aneuploidy as part of routine clinical care.

**Methods:**

A cross‐sectional survey was conducted across 6 antenatal clinics in England. Women with a high risk (≥1/150) Down syndrome screening result were offered NIPT, invasive testing, or no further testing. Pretest counselling was delivered as part of routine care by the local maternity team. Women were given a questionnaire containing a measure of informed choice immediately after pretest counselling.

**Results:**

In total, 220 of 247 women completed the questionnaire. Seventy‐six percent were judged to have made an informed choice, a significant decline from our previous study (89.0% vs 75.6%; χ^2^(2) = 20.2, P < .001). Of those making an uninformed choice, 46% had insufficient knowledge, 19% had not deliberated, and 13% had made a value‐inconsistent decision. Multivariate analysis showed women who were highly educated (OR, 4.33; 95% CI, 1.08‐17.36) or had had screening in a previous pregnancy (OR, 0.24; 95% CI, 0.90‐0.65) were significantly more likely to make an informed choice.

**Conclusions:**

The findings highlight the challenges of ensuring informed choice in routine prenatal care where NIPT is not discussed at multiple points, less time is available for counselling, and written consent is not required.

## Abstract 2

What's already known about this topic?

- Non‐invasive prenatal testing for aneuploidy is a highly accurate screening test, but concerns exist around potential routinisation.
- Previous evidence indicated high levels of informed choice are possible, but this was a tightly controlled research setting.

What does this study add?

- Non‐invasive prenatal testing can be offered within routine prenatal care in a way that facilitates high levels of informed choice.
- However, the decline in rates of informed choice compared with those in the research setting highlight the challenges of offering non‐invasive prenatal testing in routine prenatal care.

## 1 INTRODUCTION

Non‐invasive prenatal testing (NIPT) using cell‐free DNA in maternal blood is an advanced screening test, which has been shown to be highly accurate for Down syndrome with detection rates of over 99% and false positive rates less than 0.1% for singleton pregnancies.^1^ The test can also be used to screen for trisomies 13 and 18 as well as sex chromosome aneuploidies; however, detection rates are slightly lower.^1^, ^2^ The lower predictive value means that NIPT is not considered diagnostic and invasive testing is recommended to confirm positive NIPT results.^3^, ^4^ Non‐invasive prenatal testing has become widely available in recent years through the private sector,^5^ and a number of studies have been conducted to address implementation within a national prenatal care setting.^6^, ^7^, ^8^ Clinical advantages include improved accuracy rates in comparison to combined screening,^9^ reduction in the need for invasive tests that carry a small risk of miscarriage,^10^ early information about risk status, and the opportunity for early reassurance for low risk women.^11^ Nevertheless, a number of disadvantages have been identified such as the smaller diagnostic yield in comparison to invasive testing with array comparative genomic hybridisation and the potential for test failures or inconclusive results.^12^, ^13^

Empirical research has demonstrated that the test is viewed favourably, with women valuing the opportunity to have a safe, accurate test that can identify cases of Down syndrome that might otherwise be missed.^14^, ^15^, ^16^, ^17^, ^18^, ^19^ However, a number of concerns related to routinisation of testing have been raised. These include women accepting NIPT without due thought because of the ease and risk free nature of the procedure,^20^, ^21^ rapid implementation raising concerns regarding the capacity of healthcare providers to counsel women appropriately,^19^, ^22^, ^23^ NIPT being performed without women realising that they are having a screening test,^24^ and NIPT leading to societal pressures to participate in prenatal screening and stigmatisation of those who forego screening.^16^, ^25^ A key principle of prenatal testing is the promotion of reproductive autonomy by providing women and families with information to assist in pregnancy management with informed choice and informed consent procedures seen as protecting this principle.^21^ Consequently, much discussion has focused on the importance of facilitating informed choice through provision of balanced pretest information and nondirective counselling.^26^, ^27^, ^28^ In 2016, our research group developed and validated a measure of informed choice for women offered NIPT, which was included in a questionnaire assessing women's experience of testing.^29^ This work formed part of the RAPID evaluation study, which was designed to investigate implementation of NIPT into the maternity care pathway in the UK National Health Service (NHS).^30^ Using the measure, we found that 89% of women had made an informed choice.^29^

A key limitation of that informed choice study was that NIPT was offered within a highly controlled research setting where participants were given written information at multiple stages and received up to 30 minutes pretest counselling with a specifically trained research midwife who obtained written consent.^31^ At that time, we acknowledged that this degree of information and counselling may be challenging to replicate in a routine clinical setting and recommended further evaluation following NIPT implementation.^30^ In this paper, we report a follow‐up study designed to assess rates of informed choice among women offered NIPT following a high risk Down syndrome screening result (≥1.150) as part of routine NHS care.

**WHAT'S ALREADY KNOWN ABOUT THIS TOPIC?**

- Non‐invasive prenatal testing for aneuploidy is a highly accurate screening test, but concerns exist around potential routinisation.
- Previous evidence indicated high levels of informed choice are possible, but this was a tightly controlled research setting.

**WHAT DOES THIS STUDY ADD?**

- Non‐invasive prenatal testing can be offered within routine prenatal care in a way that facilitates high levels of informed choice.
- However, the decline in rates of informed choice compared with those in the research setting highlight the challenges of offering non‐invasive prenatal testing in routine prenatal care.

## 2 METHODS

National Health Service Research Ethics Committee approval was obtained for this study in February 2013 (London—Camden and Islington 13/LO/0082).

### 2.1 Participants

**Figure 1:**
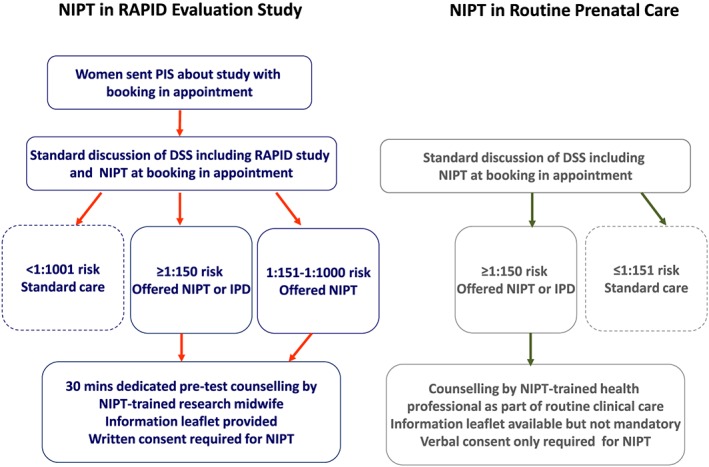
Care pathways for the delivery of NIPT [Colour figure can be viewed at http://wileyonlinelibrary.com]

Between March 1, 2015, and October 31, 2016, women with a singleton pregnancy who were identified as high risk (≥1/150) following Down syndrome screening in antenatal clinics in London and South East England were offered the option of either NIPT (for T21, T18, and T13), invasive testing (QF‐PCR on trisomies), or no further testing. Both NIPT and invasive testing were offered at no cost to parents. The cfDNA sequencing was performed by our NHS service laboratory (North East Thames Regional Genetics Service) as previously described for the research trial.^6^ For women identified as having a Down syndrome screening risk ≥1/150, a member of the local maternity care team (either a fetal medicine midwife or consultant) discussed the options available including NIPT, invasive testing, or no further investigations. This follows the model of implementation favoured by the UK National Screening Committee.^32^ Prior to the study, maternity care teams were given group training about NIPT by a member of the RAPID research team,^31^ and the NIPT information leaflet developed by the RAPID team was available to give to patients. Written consent for NIPT was not required. The care pathways for the delivery of NIPT for the both the RAPID evaluation study and this study are presented in Figure 1.

### 2.2 Information and counselling

The health professional training given to local maternity units highlighted that the information given to women during pretest counselling should include the following key points: that it is a blood test and there is no risk of miscarriage associated with the test; that it tests for Down syndrome, Edward syndrome, and Patau syndrome; that it detects around 99% of cases where the baby has Down syndrome but that there is a small chance (0.5%‐1%) of a false positive and therefore invasive testing is recommended to conform a positive result; that the NIPT result will be ready within 7 to 10 days; that in a small number of cases, the result is inconclusive or the test fails in which case a repeat NIPT will be offered; that it is less accurate than invasive testing that is over 99.9% accurate; that invasive testing carries a small risk of miscarriage (0.5%); and that invasive test results will be ready in 3 working days. The aim of the training sessions was to provide information about NIPT and answer any questions health professionals had.

### 2.3 Study procedure

The study was conducted at 6 maternity units to allow recruitment from a wide range of social and ethnic groups including a large South Asian population at 2 of the sites. Four of these units had previously recruited participants into the RAPID evaluation study; two had not. Between October 2015 and September 2016, women who were able to read and understand English were invited to take part in this study on informed choice. A consecutive sample of women were recruited across each of 6 sites. After the discussion in the clinic, potential participants were given a Participant Information Sheet about the study and a paper copy of the questionnaire (Figure S1) to complete immediately after counselling at the clinic, irrespective of what prenatal testing option they chose. Participants who were unable to complete the questionnaire at clinic were offered the option of taking it home and returning it in the freepost envelope within 1 week.

### 2.4 Questionnaire

The questionnaire included the measure of informed choice for NIPT, which comprised questions to assess knowledge, attitude, deliberation,^33^ and NIPT uptake. The development and validation of the measure are described in detail elsewhere.^29^ An informed choice was defined as one whereby the participant had good knowledge, had deliberated, and had a positive attitude and had NIPT or had a negative attitude and had declined NIPT (classified as value consistency).^29^ The questionnaire also included the Decisional Conflict Scale,^34^ the short form of the State Trait Anxiety Inventory (STAI‐6),^35^ questions to explore motivations for testing, and questions on parity and sociodemographic questions.

### 2.5 Data analysis

To assess informed choice, knowledge scores, attitude scores, and deliberation scores were combined with test behaviour. Knowledge scores were dichotomised as good or poor using a preset cut‐off of ≥9/12. Attitude scores were classified as positive, negative, or neutral with neutral responders being omitted (approach based on van den Berg et al).^29^ Deliberation scores were dichotomised as either deliberated or nondeliberated. For the purposes of the informed choice calculation, we excluded women who had declined NIPT but had opted for invasive testing. This was because whilst many of them had a positive attitude towards NIPT, they had declined the test and opted for invasive testing which, according to the definition of the measure, would have categorised them as having made an uninformed choice. However, a limitation of the measure is that it is unable to account for these more complex situations whereby a positive attitude towards NIPT is not behaviourally implemented, not because the decision is uninformed but for practical reasons such as not wanting to wait 7 to 10 days for the test result or the indication for trisomy being so strong that invasive testing was considered most appropriate. A similar approach was taken by van Schendel et al in their assessment of informed choice.^36^

The Decisional Conflict Scale score was calculated as directed by O'Connor^34^ with participants scoring ≥37.5 categorised as having decisional conflict. The 6‐item State Trait Anxiety Index was scored according to the authors' instructions^35^ with scores of ≥50 indicating elevated state anxiety. Descriptive analysis was conducted on single items. Regression analysis was conducted to determine which independent variables were significant predictors of informed choice. Religion and ethnicity were collapsed into binary variables to strengthen the analysis. A Mann‐Whitney test was used to determine differences in decisional conflict and anxiety between women making informed and uninformed decisions. A chi‐square test was used to compare rates of informed choice between the RAPID evaluation study and this study. Missing data on the knowledge scale were treated as incorrect answers. Questionnaires with ≥50% missing data were removed from the analysis. Where there was <50% missing data, missing values were replaced by imputing the mean. Analysis was performed using SPSS 22 (IBM, Chicago, Illinois).

## 3 RESULTS

### 3.1 Sample characteristics

A total of 220 of 247 women invited to take part in the study completed Q1 (89% response rate). Two questionnaires were removed due to ≥50% missing data (N = 218). Participant characteristics are presented in Table 1. The mean age was 35.7 years (range, 23‐47). One participant had opted for no further testing, and 10 had chosen invasive testing over NIPT. Mean Down risk scores did not differ significantly between women who opted for invasive testing vs NIPT (mean, 58.4 vs 73.8, respectively; *F* = 1.10, *P* = .30).

**Table 1:** Participant characteristics

Participant Characteristics	N = 218 n (%)
Maternal age—mean; range (missing 10)	35.7 y; 23‐47
Educational level (missing 7)	
No qualification	5 (2.4)
GCSE or O level	22 (10.4)
GCE, A level or similar	15 (7.1)
Vocational (BTEC/NVQ/diploma)	30 (14.2)
Degree level or above	139 (65.9)
Ethnicity (missing 15)	
White or White British	145 (71.4)
Asian or Asian British	21 (10.3)
Black or Black British	19 (8.7)
Other ethnic group	16 (7.9)
Mixed	2 (0.9)
Religious faith (missing 9)	
Yes	120 (56.6)
No	92 (43.4)
Which faith (missing 0)	
Christian	88 (73.3)
Muslim	22 (18.3)
Jewish	3 (2.5)
Other	3 (2.5)
Sikh	2 (1.7)
Hindu	1 (0.8)
Buddhist	1 (0.8)
Religiosity (missing 25)	
Very	22 (23.2)
Somewhat	57 (60.0)
Not at all	16 (16.8)
Further testing (missing 0)	
NIPT	207 (94.9)
Invasive testing	10 (4.6)
No further testing	1 (0.5)
Children (missing 5)	
Yes	133 (62.4)
No	80 (37.6)
DSS in previous pregnancy^a^	
Yes	90 (68.2)
No	36 (27.3)
Not sure	6 (4.5)
Have a child with DS (missing 5)	
Yes	3 (2.3)
No	125 (97.7)
Know anyone who has a child with DS (missing 17**)**	
Yes	50 (24.9)
No	151 (75.1)

Abbreviations: DSS = Down syndrome screening, DS = Down syndrome; NIPT, non‐invasive prenatal testing.

Note: Not all % add up to 100 due to rounding. Not all participants answered all questions, and therefore, there are some discrepancies with total numbers.

a It is not possible to work out “missing” here as some may have had a previous pregnancy that did not result in having a child presently.

### 3.2 Informed choice

Of the total sample (N = 218), 84.9% had good knowledge (n = 185); 81.7% had a positive attitude (n = 178), 12.8% (n = 28) had a negative attitude, and 5.5% had a neutral attitude (n = 12); and 93.1% had deliberated (n = 203). Of the 10 women who opted for invasive testing over NIPT, 6 had a positive attitude towards NIPT, 3 had a negative attitude, and 1 had a neutral attitude.

For the informed choice calculation, 11 questionnaires were excluded because the participant had a neutral attitude towards NIPT, 9 questionnaires were removed because the participant had opted for invasive testing, and 1 questionnaire was removed because the participant had a neutral attitude and had opted for invasive testing. The informed choice analysis therefore included 197 questionnaires and showed that 75.6% of women (n = 149) made an informed choice about NIPT; 88.8% (n = 175) had good knowledge, 95.4% (n = 188) had deliberated, and 87.3% (n = 172) made a decision that was value consistent. Of those participants that made an uninformed choice (24.4%, n = 48), 45.8% (n = 22) had insufficient knowledge, 18.8% (n = 9) had not deliberated, and 13.2% (n = 26) had made a value‐inconsistent decision (Table 2).

**Table 2:** Types of informed and uninformed choices

	Knowledge	Deliberation	Attitude	Uptake	n (%)
Informed choice	Good	Yes	Positive	Yes	149 (75.6)
	Good	Yes	Negative	No	0 (0)
Uninformed choice	Good	Yes	Negative	Yes	17 (8.6)
	Poor	Yes	Positive	Yes	16 (8.1)
	Good	No	Positive	Yes	6 (3.0)
	Poor	Yes	Negative	Yes	6 (3.0)
	Good	No	Negative	Yes	2 (1.0)
	Good	Yes	Positive	No	1 (0.5)
	Poor	No	Positive	Yes	1 (0.5)
	Good	Yes	Positive	No	0 (0)
	Good	No	Negative	No	0 (0)
	Good	No	Positive	No	0 (0)
	Good	No	Negative	Yes	0 (0)
	Poor	Yes	Positive	No	0 (0)
	Poor	No	Positive	No	0 (0)
	Poor	No	Negative	Yes	0 (0)

Univariate analysis showed that women making an informed choice were significantly more likely to be highly educated (OR, 3.37; 95% CI, 1.26‐8.97; *P* < .05), have no religious affiliation (OR, 0.43; 95% CI, 0.21‐0.88; *P* < .05), be White ethnicity (OR, 0.37; 95% CI, 0.19‐0.73; *P* < .05), and had DS screening in a previous pregnancy (OR, 0.27; 95% CI, 0.11‐0.64; *P* < .05). Multivariate analysis showed that women who were highly educated (OR, 4.33; 95% CI, 1.08‐17.36; *P* < .05) or had DS screening in a previous pregnancy (OR, 0.24; 95% CI, 0.90‐0.65; *P* < .05) were significantly more likely to make an informed choice (Table 3). We then explored the relationship between education level and the subscales of informed choice further. Analysis indicated that there was a positive association between higher education level and higher knowledge score (H(2) = 11.04, *P* = .004), whereas education was not associated with attitude or deliberation scores.

**Table 3:** Univariate and multiple logistic regression looking at factors associated with making an informed choice

	Univariate Logistic Regression **Informed Choice (N = 197)**		Multiple Logistic Regression Informed Choice (N = 172)
Variable	Made IC	OR (95%CI)	OR (95% CI)
Age			
≤34	47 (69.1%)	1	1
35‐39	63 (79.7%)	1.76 (0.83‐3.73)	1.48 (0.62‐3.53)
≥40	36 (83.7%)	2.30 (0.88‐6.00)	1.94 (0.64‐5.82)
Level of education			
GCSE or lower	11 (55.0%)	1	1
GCE or vocational	28 (73.7%)	2.29 (0.73‐7.16)	2.99 (0.86‐10.35)
Degree or above	107 (80.5%)	3.37 (1.26‐8.97)*	4.33 (1.08‐17.36)*
Ethnicity			
White	110 (82.1%)	1	1
Other	39 (62.9%)	0.37 (0.19‐0.73) *	0.53 (0.22‐1.25)
Religion			
No	70 (84.3%)	1	1
Yes	76 (69.7%)	0.43 (0.21‐0.88)*	0.57 (0.24‐1.32)
Had DS screening previously			
Yes	67 (80.7%)	1	1
No or not sure	18 (52.9%)	0.27 (0.11‐0.64)*	0.24 (0.90‐0.65)*
Not applicable	61 (82.4%)	1.12 (0.50‐2.52)	0.99 (0.37‐2.63)
Has or knows a child with DS			
Yes	38 (80.9%)	1	1
No	100 (74.6%)	0.70 (0.31‐1.59)	0.79 (0.34‐2.03)

Abbreviation: DS, Down syndrome.

* *P* < .05.

** *P* < .001.

### 3.3 Comparison with RAPID evaluation study

There was a significant decline in the rate of informed choice in this study when compared to the RAPID evaluation study (89.0% vs 75.6%; χ^2^(2) = 20.2, *P* < .001). Knowledge scores were significantly lower (95.4% vs 88.8%; χ^2^(2) = 10.4, *P* = .001), and fewer participants had a positive attitude towards NIPT (97.7% vs 87.3%; χ^2^(2) = 32.0, *P* < .001). When comparing the high risk women in this study with high risk women from the RAPID evaluation study, rates of informed choice were still significantly lower (93.5% vs 76.5%; χ^2^(2) = 19.8, *P* < .001), there were still significantly fewer women in this study with a positive attitude (97.4% vs 87.3%; χ^2^(2) = 11.5, *P* = .001), and fewer women judged to have good knowledge (96.7% vs 88.8%; χ^2^(2) = 7.5, *P* = .06); however, this was not significant. Deliberation scores were not significantly different when comparing women in this study with the total RAPID evaluation study sample nor high risk women only (93.9% vs 95.4%, χ^2^(2) = 0.6, *P* = .43 and 97.4% vs 95.4%, χ^2^(2) = 0.9, *P* = .343).

### 3.4 Decisional conflict and state‐trait anxiety

Decisional conflict occurred in 6.6% (n = 13) of cases. Women who made an uninformed decision were significantly more likely to experience decisional conflict than women who made an informed decision (Mdn, 19.53 vs 1.56, respectively; *U* = 1980, *P* < .001). Anxiety was found to be elevated in 62.6% (n = 119) of cases. Anxiety scores were not found to have a significant impact on informed decision‐making (Mdn 50.00 uninformed choice vs Mdn 53.3 informed choice; *U* = 2866, *P* = .217). There were no significant differences between the women in this study and the high risk women in the RAPID evaluation study when we looked at decisional conflict (3.0% vs 6.6%; χ^2^(2) = 2.4, *P* = .12) and anxiety (54.5% vs 60.4%; χ^2^(2) = 1.2, *P* = .261).

### 3.5 Motivations for accepting or declining NIPT

Of those women who opted for NIPT, the most frequently cited reason was “for reassurance that the baby doesn't have Down syndrome” (28.1%) followed by “to help me make a decision about whether or not continue with the pregnancy” (21.9%). Of those women who chose invasive testing over NIPT, the most frequently cited reason was because they would “get the results more quickly” (46.6%). Only one participant declined any further testing, the reason being “I would never terminate an affected pregnancy so there would be no point taking the test” (Table 4).

**Table 4:** Motivations for accepting or declining non‐invasive prenatal testing (NIPT)

Motivations for choosing NIPT	Total N = 338
For reassurance that my baby doesn't have Down syndrome	n = 95 (28.1%)
To help me make a decision about whether or not to continue with the pregnancy	n = 74 (21.9%)
I would want as much information about the baby as possible	n = 55 (16.3%)
So I can plan and prepare for the birth of a baby with Down's syndrome	n = 35 (10.4%)
Because there is no risk to the baby	n = 21 (6.2%)
To avoid having a child with Down's syndrome	n = 12 (3.6%)
Because it was offered to me as part of my antenatal care	n = 10 (1.2%)
Other	n = 3 (0.9%)
Because my partner or family would want me to	n = 1 (0.3%)

Motivations for choosing invasive testing	Total N = 15
I will get the results more quickly	n = 7 (46.6%)
It is more accurate than NIPT	n = 4 (26.7%)
The indication for Down syndrome or another chromosomal abnormality was so strong that I chose invasive testing	n = 4 (26.7%)
Other	n = 0 (0.0%)

Note: N = total number of responses. Participants were allowed to select up to 2 responses for the motivations to accept NIPT.

## 4 DISCUSSION

Many of the ethical concerns raised about NIPT relate to routinisation of testing and erosion of informed choice.^20^, ^21^, ^23^, ^37^ Our study indicates that even when NIPT is offered as part of routine clinical practice, it is possible to achieve high rates of informed decision‐making amongst women who choose to have NIPT. Most women in this study had good knowledge about NIPT, had deliberated, and had positive attitudes towards NIPT. These findings concur with our previous work^29^ and other recently published studies assessing informed choice in a research setting such as the study published in the Netherlands where 78% of women were found to have made an informed choice about NIPT using a similar measure assessing knowledge, attitudes, and uptake,^36^ as well as research examining patient understanding of NIPT.^38^, ^39^ Nevertheless, the rate of informed choice was found to be significantly lower in this study compared to our previous study.^29^ This is perhaps not surprising given that in our previous study, NIPT was being offered within a highly controlled research environment with additional time provided for pretest counselling, clear signposting of NIPT at multiple time points, and women were asked to sign a consent form. The findings from our current study might be considered to give a more realistic representation of informed choice amongst women who are high risk and making decisions about NIPT. The finding that rates of informed choice were lower once NIPT was offered by the clinical team, without additional input from researchers or the need to sign a consent form, highlights the challenges of ensuring high quality pretest counselling in the context of routine prenatal care.

Our data, along with others,^36^, ^38^, ^39^ indicate that particular attention should be given to women with lower education and/or health literacy levels as they are less likely to have sufficient knowledge to make informed decisions about NIPT and underscores the importance of developing novel tools to support this group of women. The development of interactive decision aids to complement written and verbal information might be one potential solution. Interactive computer aids have been found to improve patient knowledge in a number of studies focused on prenatal testing.^40^, ^41^ Alternatively, an informational film could be developed and piloted. This approach was found to enhance knowledge and informed decision‐making around Down syndrome screening in a study conducted in Sweden.^42^

As has been identified in other studies looking at informed decision‐making in pregnancy,^36^, ^43^ decisional certainty was found to be positively associated with an informed decision, supporting the use of the Decisional Conflict Scale as a good indicator of informed decision‐making and underscoring that informed choice is associated with better psychological outcomes.^44^ Decisional conflict, the extent to which a person feels uncertain, unclear about personal values, and unsupported in decision‐making, has been associated with decisional regret,^45^which could potentially have serious consequences for women making important decisions in pregnancy. Further research to address causes of decisional conflict and ways in which these women may benefit from additional decision support to reduce their uncertainty would be valuable.

A number of recently published papers have demonstrated that patients may underestimate the limitations of NIPT, in particular the potential for a false positive or inconclusive result.^38^, ^39^ These concerns are supported to some extent by our results as the question answered incorrectly most frequently on the knowledge scale related to the potential for an inconclusive NIPT result (Table S1). Inconclusive NIPT results may occur due to low fetal fraction or sequencing failures and have been reported as occurring as frequently as 6% of cases.^12^ Recent publications also suggest that patients who receive an inconclusive NIPT result may be at increased risk of aneuploidy.^9^ These findings highlight the importance of pretest counselling in addressing any misconceptions about the test and ensuring patients understand the potential for an inconclusive result to reduce the chance of decisional regret later on.

In this study, NIPT was offered as a contingent test to women with a high risk result from the combined screening test, in line with the model the UK National Screening Committee plan for NHS implementation in 2018.^32^ However, NIPT is increasingly being made available to the general obstetric population in the UK through private practice as a first line test. The importance of a multistep deliberative process facilitated through contingent testing has been identified previously in studies of women's experience of being offered NIPT.^15^ However, as the cost of sequencing falls and NIPT testing gets cheaper, there is the potential to use it as a first line screening test. Given that rates of informed choice fell when offered in routine clinical practice, we will need to carefully re‐evaluate this in settings where NIPT is offered as a first line test.

We used a measure of informed choice that has previously been validated amongst women considering NIPT as a second screening test and had a high response rate. A strength of this measure is that it is sensitive enough to pick up differences between different settings. However, a limitation relates to the attitude scale not being sophisticated enough to pick up informed decision‐making in certain circumstances, such as where a positive attitude is not behaviourally implemented because an alternative testing option is preferred. By excluding women who chose invasive testing from the informed choice calculation, we may have artificially increased the percentage of women making informed choices. Further development of the attitude scale to address this limitation is required.

The main limitation of this study is that we did not receive any questionnaires from NIPT decliners. We therefore cannot comment on whether they had made an informed choice to decline NIPT. This was also the case in our previous study, where only 13 of 585 participants had declined further tests. In this study, training was available to the local maternity care teams, and the intention was to include all health professionals who would speak to women about NIPT, including the midwives who would discuss the test at booking and those who would offer NIPT and give results. We do not, however, know what cascade training was done with health professionals who did not attend training. However, this does reflect reality of how a busy maternity department works. Training was provided by the RAPID research team, and 4 of the 6 units had participated in the RAPID evaluation study, which could be perceived as an inherent bias in the study. Our sample predominantly comprised older, well‐educated women; however, this probably reflects the fact that older women are more likely to be at increased risk and be highly educated as they have delayed child bearing for educational or vocational reasons. Finally, in our study, we found that 62.6% of women had elevated anxiety scores at the time of testing. This finding is in line with other studies looking at anxiety in women identified as high risk through screening.^46^ Nevertheless, the absence of a baseline anxiety assessment is a limitation as we are unable to determine whether these women were anxious prior to testing or whether anxiety increased as a result of screening.

## 5 CONCLUSION

Non‐invasive prenatal testing is set to become part of routine care in the UK NHS imminently. Maintaining high levels of informed choice will be very dependent on effective training of health professionals to ensure they can provide up‐to‐date unbiased information and also have the confidence and skills to support parents to discuss prenatal testing options in a way that reflects their patients' values and beliefs. Recent guidelines emphasise the importance of having pretest face‐to‐face conversations with patients about their values regarding termination and pregnancy planning to help direct and personalise counselling as well as ensuring patients are clear that screening and testing are optional.^47^ Current research shows it is possible to achieve high levels of informed decision‐making for NIPT, but given the possibility of NIPT as a first line screening test continued research in this area is important.

## CONFLICT OF INTEREST

The authors declare no conflict of interest.

## FUNDING STATEMENT

This manuscript presents independent research funded by the National Institute for Health Research (NIHR) under the Programme Grants for Applied Research programme (RP‐PG‐0707‐10107) (the ‘RAPID’ project). LSC is partially funded by the NIHR Biomedical Research Centre at Great Ormond Street Hospital.

## Supporting information


**Figure S1.** Questionnaire T1: An evaluation of NIPT for aneuploidy in an NHS setting

**Table S1:** Knowledge Measure
