# Supplementary material for: Offering non‐invasive prenatal testing as part of routine clinical service. Can high levels of informed choice be maintained?
Source: Prenat Diagn. 2017 Oct 17;37(11):1130–7. doi: 10.1002/pd.5154 (PMC5969260; doi:10.1002/pd.5154)
Supplement: Supplementary file 1 — Figure S1. Questionnaire T1: An evaluation of NIPT for aneuploidy in an NHS setting [file PD-37-1130-s001.doc]

Questionnaire T1: An evaluation of NIPT for aneuploidy in an NHS setting

Principal Researcher: Professor Lyn Chitty

Version 2: 1^st^ July 2015

Recruiting centre:

Participant identification number:

Date completed:

**An evaluation of NIPT for aneuploidy in an NHS setting**

**What is the purpose of the study?**

To investigate the experience of women offered new non-invasive prenatal tests (NIPT) for Down’s syndrome and some other chromosomal conditions during pregnancy.

**Do I have to take part?**

Participation in this study is completely voluntary; you do not have to take part if you do not wish to. You are free to withdraw at any time.

**Will my answers affect my healthcare?**

The choices you make on this questionnaire will not affect your healthcare in any way.

**Will my participation be kept confidential?**

Yes. All responses will be strictly confidential.

**Who is organising and funding the research?**

The research is organised by the Research Teams at Great Ormond Street Hospital and UCL Institute of Child Health. The study is funded by the National Institute for Health Research.

**Who has reviewed the study?**

This study has been reviewed and given favourable opinion by the Camden and Islington National Research Ethics Service Committee.

**Where can I get further information?**

If you have any questions about the study or the questionnaire please speak to a member of the research team. Phone: 020 78298653.

If you would like to speak to someone external to the study for information and support around prenatal testing you can contact the Antenatal Results and Choices (ARC) helpline. Phone: 020 7713 7486

**What should I do when I complete the questionnaire?**

Please hand completed paper questionnaires back to the midwife/researcher or receptionist in the Antenatal Unit. If you don’t complete your questionnaire today you can pick up a freepost envelope at reception.

**A. Answer the following questions one by one. Please do not read through to the end of this section before you begin as this may give some of the answers away and not reflect your true understanding of the test. Please do not go back and change responses once you have completed the questions.**

| **1. Which of these conditions does** non-invasive prenatal test (NIPT) test the baby for? (tick one answer only)   | Spina bifida |  | | --- | --- | | Anaemia |  | | Down’s syndrome |  | | Down’s syndrome (and 2 rarer chromosome conditions Edward’s and Patau syndrome) |  | | All known genetic conditions |  | | Not sure |  | |
| --- | --- | --- | --- | --- | --- | --- | --- | --- | --- | --- | --- | --- |
|  |
| 2. How is NIPT done? (tick one answer only)   | Saliva test from the mother |  | | --- | --- | | Urine test from the mother |  | | Blood test from the mother |  | | Invasive test taking amniotic fluid from around the baby |  | | Not sure |  | |
| **3. What does a predicted to be affected NIPT result mean**? (tick one answer only)   | The baby definitely has the condition |  | | --- | --- | | It is highly likely that the baby has the condition, but invasive testing is needed to confirm the diagnosis |  | | Not sure |  |   **4. What does a highly unlikely to be affected NIPT result** mean? (tick one answer only)   | The baby definitely does not have the condition |  | | --- | --- | | It is highly unlikely that the baby has the condition, but as the test is not 100% accurate there is a very small chance the result is wrong |  | | Not sure |  |   **5.** How does NIPT compare with standard Down’s syndrome screening tests (ultrasound scan and/or blood test from the mother) currently offered during pregnancy? (tick one answer only)   | It is less accurate |  | | --- | --- | | It has the same accuracy |  | | It is more accurate |  | | Not sure |  |   **6.** How safe is NIPT (tick one answer only)   | There is no risk to you or the baby |  | | --- | --- | | There is a risk of miscarriage |  | | Not sure |  |   **7**. How long does it take to get an NIPT result? (tick one answer only)   | The result will be available immediately after the blood is taken |  | | --- | --- | | It takes 24 hours to get a result |  | | It takes 7-10 working days to get a result |  | | Not sure |  |   **8. Will you always get a test result?** |
| | Yes, it is certain that you will receive a test result |  | | --- | --- | | No, in a small number of cases the laboratory can’t give a result and the test can be repeated |  | | Not sure |  | |
|  |
| **9. How safe are invasive tests (amniocentesis or CVS)? (tick one answer only)**   | There are no risks to you or the baby |  | | --- | --- | | There is a small (around 1%) risk of miscarriage |  | | There is a high (20%) risk of miscarriage |  | | None of these |  | | Not sure |  | |  |  | |
| **10. If it is confirmed that your baby definitely does have the condition, what will you be offered? (tick all that apply)**   | Immediate treatment for the baby |  | | --- | --- | | Support to prepare for a baby with the condition |  | | The option of terminating the pregnancy if you want to |  | | None of these |  | | Not sure |  | |
| **11**. Do you have to take any of these tests? (tick one answer only)   | Yes, all women have to take these tests in pregnancy |  | | --- | --- | | No, it is my choice whether or not to take these tests |  | | Not sure |  |   **12. What is Down’s syndrome? (tick one answer only)**   | A life-long condition that causes learning difficulties |  | | --- | --- | | A condition that can be cured by surgery |  | | A condition that children grow out of |  | | Not sure |  | |
|  |
|  |
| **B. For each of the following five questions, please circle the number from 0 to 4 on the scale that best describes how you feel at the moment.** |
| **13. For me, having NIPT would be:**   | Beneficial | 0 | 1 | 2 | 3 | 4 | Harmful | | --- | --- | --- | --- | --- | --- | --- |   **14.** **For me, having NIPT would be:**   | Important | 0 | 1 | 2 | 3 | 4 | Unimportant | | --- | --- | --- | --- | --- | --- | --- |   **15.** **For me, having NIPT would be:**   | A good thing | 0 | 1 | 2 | 3 | 4 | A bad thing | | --- | --- | --- | --- | --- | --- | --- |   **16.** **For me, having NIPT would be:**   | Reassuring | 0 | 1 | 2 | 3 | 4 | Not reassuring | | --- | --- | --- | --- | --- | --- | --- |   **17.** **For me, having NIPT would be:**   | Desirable | 0 | 1 | 2 | 3 | 4 | Undesirable | | --- | --- | --- | --- | --- | --- | --- | |

**C. Uptake**

**18. Which option did you choose regarding NIPT?**

| 1. I chose to have NIPT |  |
| --- | --- |
| 1. I chose to have no further testing |  |
| 1. I chose to have invasive testing only |  |

**D. Please reflect on the decision you made whether or not to have non-invasive prenatal testing. Please show how strongly you agree or disagree with these statements by circling a number from 0 (strongly agree) to 4 (strongly disagree) which best fits your views about your decision.**

| **19. I have tried to consider all the different options**   | Strongly agree | 0 | 1 | 2 | 3 | 4 | Strongly disagree | | --- | --- | --- | --- | --- | --- | --- |   **20. I have imagined how I would feel if I did not have NIPT**   | Strongly agree | 0 | 1 | 2 | 3 | 4 | Strongly disagree | | --- | --- | --- | --- | --- | --- | --- |   **21. I have imagined how I would feel if I did have NIPT**   | Strongly agree | 0 | 1 | 2 | 3 | 4 | Strongly disagree | | --- | --- | --- | --- | --- | --- | --- |   **22. I have tried to think through the consequences of not accepting NIPT**   | Strongly agree | 0 | 1 | 2 | 3 | 4 | Strongly disagree | | --- | --- | --- | --- | --- | --- | --- |   **23. I have tried to think through the consequences of accepting NIPT**   | Strongly agree | 0 | 1 | 2 | 3 | 4 | Strongly disagree | | --- | --- | --- | --- | --- | --- | --- |   **24. I have made a (mental) list of the pros and cons of NIPT**   | Strongly agree | 0 | 1 | 2 | 3 | 4 | Strongly disagree | | --- | --- | --- | --- | --- | --- | --- | |
| --- | --- | --- | --- | --- | --- | --- | --- | --- | --- | --- | --- | --- | --- | --- | --- | --- | --- | --- | --- | --- | --- | --- | --- | --- | --- | --- | --- | --- | --- | --- | --- | --- | --- | --- | --- | --- | --- | --- | --- | --- | --- | --- |

**E. Please now consider the different options you were presented with regarding prenatal testing. These included accepting NIPT, declining NIPT, or invasive testing (a CVS or amniocentesis). Considering the option you chose, please answer the following questions.**

**25. I know which options are available to me**

| Strongly agree | 0 | 1 | 2 | 3 | 4 | Strongly disagree |
| --- | --- | --- | --- | --- | --- | --- |

**26. I know the benefits of each option**

| Strongly agree | 0 | 1 | 2 | 3 | 4 | Strongly disagree |
| --- | --- | --- | --- | --- | --- | --- |

**27. I know the risks and side effects of each option**

| Strongly agree | 0 | 1 | 2 | 3 | 4 | Strongly disagree |
| --- | --- | --- | --- | --- | --- | --- |

**28**. I am clear about which benefits matter most to me

| Strongly agree | 0 | 1 | 2 | 3 | 4 | Strongly disagree |
| --- | --- | --- | --- | --- | --- | --- |

**29. I am clear about which risks and side effects matter most to me**

| Strongly agree | 0 | 1 | 2 | 3 | 4 | Strongly disagree |
| --- | --- | --- | --- | --- | --- | --- |

**30. I am clear about whether the benefits or potential risks are more important to me**

| Strongly agree | 0 | 1 | 2 | 3 | 4 | Strongly disagree |
| --- | --- | --- | --- | --- | --- | --- |

**31. I have enough support from others to make a choice**

| Strongly agree | 0 | 1 | 2 | 3 | 4 | Strongly disagree |
| --- | --- | --- | --- | --- | --- | --- |

**32.** I am choosing without pressure from others

| Strongly agree | 0 | 1 | 2 | 3 | 4 | Strongly disagree |
| --- | --- | --- | --- | --- | --- | --- |

**33.** I have enough advice to make a choice

| Strongly agree | 0 | 1 | 2 | 3 | 4 | Strongly disagree |
| --- | --- | --- | --- | --- | --- | --- |

**34.** I am clear about the best choice for me

| Strongly agree | 0 | 1 | 2 | 3 | 4 | Strongly disagree |
| --- | --- | --- | --- | --- | --- | --- |

**35.** I feel sure about what to choose

| Strongly agree | 0 | 1 | 2 | 3 | 4 | Strongly disagree |
| --- | --- | --- | --- | --- | --- | --- |

**36.** This decision is easy for me to make

| Strongly agree | 0 | 1 | 2 | 3 | 4 | Strongly disagree |
| --- | --- | --- | --- | --- | --- | --- |

**37.** I feel I have made an informed choice

| Strongly agree | 0 | 1 | 2 | 3 | 4 | Strongly disagree |
| --- | --- | --- | --- | --- | --- | --- |

**38.** My decision shows what is important to me

| Strongly agree | 0 | 1 | 2 | 3 | 4 | Strongly disagree |
| --- | --- | --- | --- | --- | --- | --- |

**39.** I expect to stick with my decision

| Strongly agree | 0 | 1 | 2 | 3 | 4 | Strongly disagree |
| --- | --- | --- | --- | --- | --- | --- |

**40. I am satisfied with my decision**

| Strongly agree | 0 | 1 | 2 | 3 | 4 | Strongly disagree |
| --- | --- | --- | --- | --- | --- | --- |

| **F. A number of statements which people have used to describe themselves are given below. Read each statement and then circle the most appropriate number to indicate how you feel right now, regarding your pregnancy. There are no right or wrong answers. Do not spend too much time on any one statement but give the answer which seems to describe your present feelings best.** | | | | |
| --- | --- | --- | --- | --- |
|  |  |  |  |  |
|  | Not at all | Somewhat | Moderately | Very much |
| **41. I feel calm** | 1 | 2 | 3 | 4 |
| **42. I am tense** | 1 | 2 | 3 | 4 |
| **43. I feel upset** | 1 | 2 | 3 | 4 |
| **44. I am relaxed** | 1 | 2 | 3 | 4 |
| **45. I feel content** | 1 | 2 | 3 | 4 |
| **46. I am worried** | 1 | 2 | 3 | 4 |

**G. Reasons behind your decision to accept or decline NIPT**

**If you accepted NIPT please go to question 47. If you declined NIPT please go to question 48.**

**47. Which statement(s) best matches your reason for accepting NIPT? *(tick up to two responses)***

| So I can plan and prepare for the birth of a baby with Down’s syndrome |  |
| --- | --- |
| To help me make a decision about whether or not to continue with the pregnancy |  |
| For reassurance that my baby doesn’t have Down’s syndrome |  |
| To avoid having a child with Down’s syndrome |  |
| I would want as much information about the baby as possible |  |
| Because there is no risk to the baby |  |
| Because my partner or family would want me to |  |
| Because it was offered to me as part of my antenatal care |  |
| Other: ___________________________________________________________________ |  |

**If you declined NIPT but had invasive testing please answer question 48:**

**48.** **Which statement(s) best matches your reason for having invasive testing? *(tick up to two responses)***

| It is more accurate than NIPT |  |
| --- | --- |
| I will get the results more quickly |  |
| The indication for Down syndrome or another chromosomal abnormality was so strong that I chose invasive testing |  |
| Other:_________________________________________________________________________ |  |
|  |  |

**If you declined any further testing please answer question 49**

**49. Which statement(s) best matches your reason for declining any further testing? *(tick up to two responses)***

| I would never terminate an affected pregnancy so there would be no point taking the test |  |
| --- | --- |
| I would not want to have to make a decision about whether to terminate the pregnancy |  |
| It would cause a lot of anxiety if the baby was found to be affected |  |
| I felt sufficiently reassured by the screening result |  |
| My partner or family would not want me to take the test |  |
| I would prefer not to know |  |
| Other________________________________________________________________________ |  |

**H. Some questions about you**

**50. How old are you? .............**

| **52. Which of the following best describes you?** | |
| --- | --- |
| **Asian or Asian British** |  |
| **Black or Black British** |  |
| Mixed |  |
| White or White British |  |
| Other ethnic group: _____________ |  |

| **51. What is your highest educational qualification?** | |
| --- | --- |
| No qualification |  |
| GCSE or O level |  |
| GCE, A-level or similar |  |
| Vocational (BTEC/NVQ/Diploma) |  |
| Degree level or above |  |

| **53. Do you have a religious faith?** | Yes |  | No |  |
| --- | --- | --- | --- | --- |

| **54. If YES, which faith?** | |
| --- | --- |
| **Christian** |  |
| **Muslim** |  |
| Jewish |  |
| Hindu |  |
| Buddhist |  |
| Sikh |  |
| Other: __________________ |  |

| **55. If YES, how religious are you?** | | |
| --- | --- | --- |
| **Not at all** |  |  |
| **Somewhat** |  |  |
| Very |  |  |

| **56. What was your Down syndrome screening result? E.g. 1 in 30; 1 in 100**  **1 in ………………………………** |
| --- |

| **57. Do you have children?** | | | |
| --- | --- | --- | --- |
| Yes |  | Go to Q58 |  |
| No |  | Go to Q64 |  |

| **58. Did you have a screening test for**  **Down’s syndrome in any of those pregnancies?** | | | |
| --- | --- | --- | --- |
| **Yes** |  | **Go to Q59** |  |
| **No** |  | **Go to Q62** |  |
| **Not sure** |  | **Go to Q63** |  |

| **59. Were you found to be high risk in any of those pregnancies?** | | | |
| --- | --- | --- | --- |
| **Yes** |  | **Go to Q60** |  |
| **No** |  | **Go to Q63** |  |
| **Not sure** |  | **Go to Q63** |  |

**60. Did you have an invasive test?**

| **61. What was the outcome?** | |  |
| --- | --- | --- |
| Diagnosis of Down’s syndrome |  | Go to Q63 |
| Diagnosis of another condition |  | Go to Q63 |
| Normal result |  | Go to Q63 |
| Miscarriage as a result of invasive test |  | Go to Q63 |
| Not sure |  | Go to Q63 |

**(CVS or amniocentesis)**

| Yes |  | Go to Q61 |
| --- | --- | --- |
| No |  | Go to Q63 |
| Not sure |  | Go to Q63 |

| | **62. If you declined a screening test for Down’s syndrome in a previous pregnancy, what was the reason why? *(tick up to two responses)*** |  | | --- | --- | | I would never terminate an affected pregnancy so there was no point taking the screening test |  | | I would not have chosen to have an invasive test and put my pregnancy at risk |  | | It would have caused a lot of anxiety if I found out I was high risk |  | | I did not want to know and then have to make a decision about what to do next |  | | Screening didn’t give me a definite result |  | | My partner or family did not want me to |  | | I preferred not to know |  | | Other: ____________________________________________________________________________ |  |  | **63. Do you have a child with Down’s syndrome?** | | | | --- | --- | --- | | Yes |  |  | | No |  |  |  | **64. Do you know anyone who has a child with Down’s syndrome?** | | | | --- | --- | --- | | Yes |  |  | | No |  |  | |
| --- | --- | --- | --- | --- | --- | --- | --- | --- | --- | --- | --- | --- | --- | --- | --- | --- | --- | --- | --- | --- | --- | --- | --- | --- | --- | --- | --- | --- | --- | --- | --- | --- | --- | --- | --- | --- |

**Thank you for completing the questionnaire.**

✂……………………………………………………………………………………………………………

As part of this study we have a second questionnaire that we will send out in 4-8 weeks time. This second questionnaire is much shorter. Ideally, we would like to send this to you by email. If you would be willing to complete the second questionnaire, please leave your email address below. If you would prefer us to send it by post, please leave your postal address instead:

Email/Address: _________________________________________________________________________
