# Supplementary material for: Offering non‐invasive prenatal testing as part of routine clinical service. Can high levels of informed choice be maintained?
Source: Prenat Diagn. 2017 Oct 17;37(11):1130–7. doi: 10.1002/pd.5154 (PMC5969260; doi:10.1002/pd.5154)
Supplement: Supplementary file 2 — Table S1: Knowledge Measure [file PD-37-1130-s002.docx]

| Table S1: Knowledge Measure | |
| --- | --- |
| **Question** | Response **N=218** |
| **Which of these conditions does non-invasive prenatal test (NIPT) test the baby for?**  Spina bifida  Anaemia  Down’s syndrome  Down’s syndrome (and 2 rarer chromosome conditions Edward’s and Patau)  All known genetic conditions  Not sure | 2 (0.9%)  1 (0.5%)  77 (35.8%)  127 (59.1%)  3 (1.4%)  5 (2.3%) |
| **How is NIPT done?**  Saliva test from the mother  Urine test from the mother  Blood test from the mother  Invasive test taking amniotic fluid from around the baby  Not sure | 0 (0%)  4 (1.8%)  207 (95%)  5 (2.3%)  2 (0.9%) |
| **What does a predicted to be affected NIPT result mean?**  The baby definitely has the condition  It is highly likely that the baby has the condition, but invasive testing is needed to confirm the diagnosis  Not sure | 4 (1.8%)  194 (89.4%)  19 (8.8%) |
| **What does a highly unlikely to be affected NIPT result mean?**  The baby definitely does not have the condition  It is highly unlikely that the baby has the condition, but as the test is not 100% accurate there is a very small chance the result is wrong  Not sure | 8 (3.7%)  198 (92.1%)  9 (4.2%) |
| How does NIPT compare with standard Down’s syndrome screening tests (ultrasound scan and/or blood test from the mother) currently offered during pregnancy?  It is less accurate  It has the same accuracy  It is more accurate  Not sure | 11 (5.1%)  8 (3.7%)  185 (85.6%)  12 (5.6%) |
| **How safe is NIPT?**  There is no risk to you or the baby  There is a risk of miscarriage  Not sure | 203 (93.5%)  5 (2.3%)  9 (4.1%) |
| **How long does it take to get an NIPT result?**  The result will be available immediately after the blood is taken  It takes 24 hours to get a result  It takes 7-10 working days to get a result  Not sure | 1 (0.5%)  3 (1.4%)  209 (95.9%)  5 (2.3%) |
| **Will you always get a test result?**  Yes, it is certain that you will receive a test result  No, in a small number of cases the laboratory can’t give a result and the test can be repeated  Not sure | 56 (25.8%)  145 (66.8%)  16 (7.4%) |
| **How safe are invasive tests (amniocentesis or CVS)?**  There are no risks to you or the baby  There is a small (around 1%) risk of miscarriage  There is a high (20%) risk of miscarriage  None of these  Not sure | 22 (10.1%)  178 (81.7%)  8 (3.7%)  0 (0%)  10 (4.6%) |
| If it is confirmed that your baby definitely does have the condition, what will you be offered?*  Immediate treatment for the baby  Support to prepare for a baby with the condition  The option of terminating the pregnancy if you want to  None of these  Not sure | 10 (3.1%)  128 (39.3%)  147 (45.1%)  0 (0%)  41 (12.6%) |
| **Do you have to take any of these tests?**  Yes, all women have to take these tests in pregnancy  No, it is my choice whether or not to take these tests  Not sure | 8 (3.7%)  206 (94.5%)  4 (1.8%) |
| **What is Down’s syndrome?**  A life-long condition that causes learning difficulties  A condition that can be cured by surgery  A condition that children grow out of  Not sure | 216 (99.1%)  0 (0%)  1 (0.5%)  1 (0.5%) |

*Participants could tick up to two responses
